# Paradigm shifts in neonatal hypoxic-ischemic encephalopathy therapeutics: a four-decade bibliometric exploration of emerging therapeutic dimensions (1985–2024)

**DOI:** 10.3389/fped.2025.1611345

**Published:** 2025-07-28

**Authors:** Juntao Shu, Ling Liu, Mei Yuan, Mingbiao Ma, Jingjing Yang

**Affiliations:** ^1^ Department of Neonatology, The Children’s Hospital of Kunming City, The Affiliated Children’s Hospital of Kunming Medical University, Kunming, Yunnan, China; ^2^ Department of Clinical Laboratory, The Children’s Hospital of Kunming City, The Affiliated Children’s Hospital of Kunming Medical University, Kunming, Yunnan, China; ^3^ Department of Geriatric, The First People’s Hospital of Yunnan Province, The Affiliated Hospital of Kunming University of Science and Technology, Kunming, Yunnan, China

**Keywords:** therapeutic hypothermia, hypoxic-ischemic encephalopathy, biomarkers, signaling pathways, emerging treatments, bibliometric analysis

## Abstract

**Aim:**

Neonatal hypoxic-ischemic encephalopathy (HIE) remains a significant cause of neonatal morbidity and mortality worldwide, necessitating the exploration of effective therapeutic interventions. Current treatment strategies primarily involve therapeutic hypothermia (TH); however, its efficacy remains inconsistent. Research topics and trends in this area remain unclear as well. This study aimed to identify key research areas, collaboration networks, and emerging trends using bibliometric analysis tools.

**Methods:**

A comprehensive analysis was conducted on 1,165 records from the Web of Science Core Collection (WoSCC) database. Various bibliometric techniques were employed, including coauthor analysis, co-occurrence analysis, co-citation analysis, reference clustering, and topic modeling, to visualize the knowledge structure and research dynamics in the HIE field.

**Results:**

The findings revealed extensive collaboration networks among authors, institutions, and countries, highlighting global efforts to address this critical neonatal condition. Recent trends identified key research areas, including TH, mild systemic hypothermia, oxidative stress, biomarkers and signaling pathways, which are essential for advancing the understanding and treatment of HIE.

**Conclusion:**

This study provides valuable insights into the current TH for neonates with HIE. Future research should focus on optimizing treatment approaches and evaluating long-term efficacy to enhance clinical applications.

## Highlights

• Bibliometric analysis of 1,165 studies reveals global collaboration networks and emerging HIE research trends, including therapeutic hypothermia (TH) optimization.
• Key focuses: pathogenesis, therapeutic hypothermia and emerging treatments.
• Future priorities: refining TH protocols and evaluating long-term neurodevelopmental outcomes to improve clinical translation.

## Introduction

Neonatal hypoxic-ischemic encephalopathy (HIE) is a form of brain damage resulting from insufficient oxygen supply or reduced blood flow during birth, commonly affecting newborns, particularly those experiencing prenatal distress. The incidence of HIE among newborns is estimated to be approximately one to six cases per 1,000 live births, with rates reaching 10–20 cases per 1,000 live births in low-income countries (1). The pathogenesis of HIE is complex, involving multiple pathophysiological processes, including hypoxia, reperfusion injury, inflammation, oxidative stress, and apoptosis (2).

Currently, effective treatment options for HIE remain limited (3). Therapeutic hypothermia (TH) is the standard treatment for moderate-to-severe HIE; however, its efficacy varies, and some newborns develop brain damage despite treatment (4). TH functions by slowing metabolic rates and reducing cell mortality, thereby protecting brain tissue to a certain extent. However, TH alone is insufficient to prevent long-term nerve damage; therefore, researchers are exploring other treatments in combination with TH. New biomarkers and emerging treatments have become the focus of research.

The current status and hotspots of TH treatment in HIE have not been analyzed bibliometrically, and research topics and trends in this area remain unclear. The aim of this study was to explore the application and research trends of TH in neonates with HIE using bibliometric kits such as CiteSpace, VOSviewer, and R software. By analyzing literature data, we aimed to identify key research areas, collaboration networks, and emerging trends in the field.

Our study involved a comprehensive analysis of 1,165 records from the Web of Science Core Collection (WoSCC). Various bibliometric techniques, including coauthor analysis, co-occurrence analysis, co-citation analysis, reference clustering, and topic modeling, were employed to visualize the knowledge structure and research dynamics of HIE.

## Methods

### Data collection

**Figure 1:**
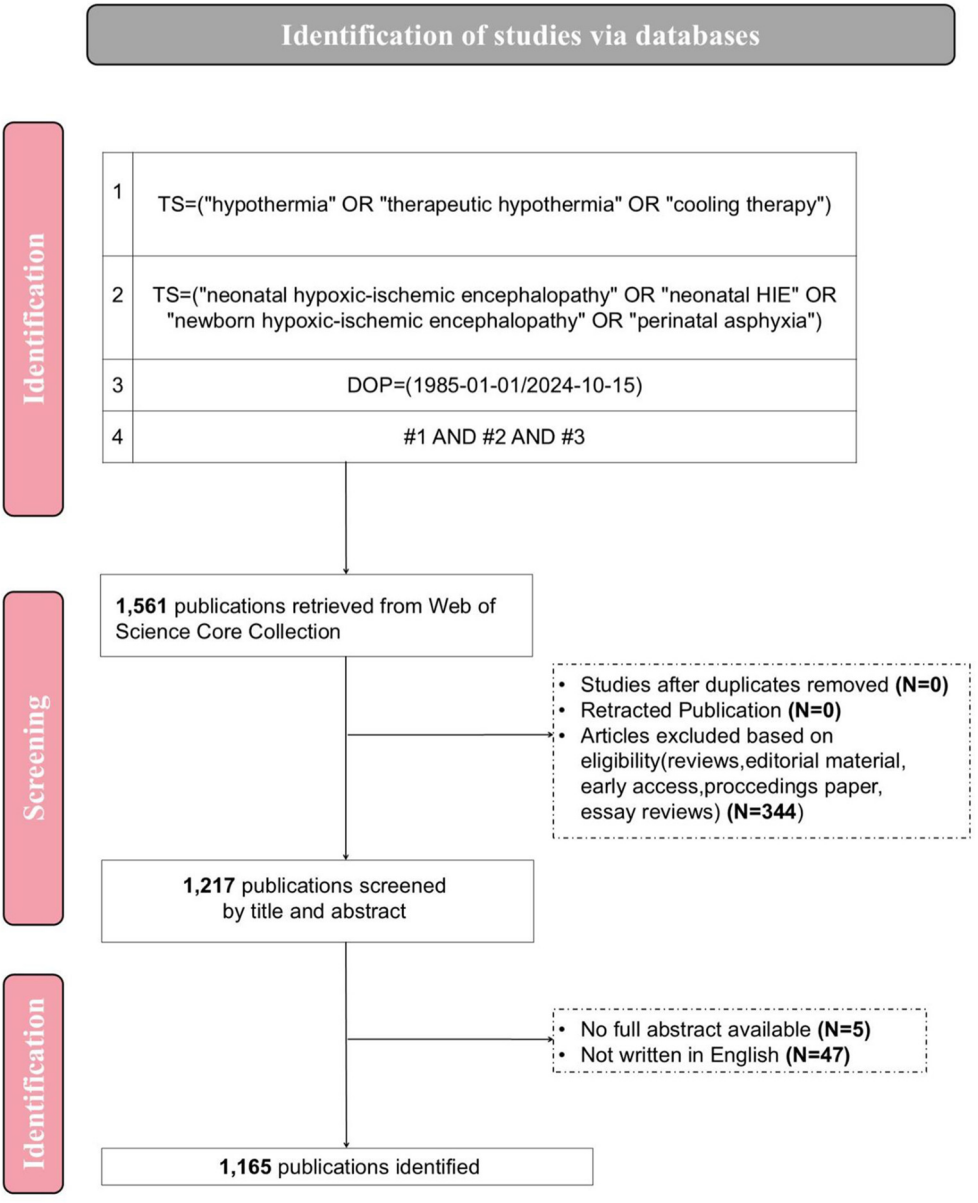
Search strategy and analysis flowchart for therapeutic hypothermia in neonatal hypoxic ischemic encephalopathy.

A comprehensive literature search of the WoSCC database was conducted on October 15, 2024. The primary search terms included “hypothermia” OR “therapeutic hypothermia” OR “cooling therapy” OR “neonatal hypoxic-ischemic encephalopathy” OR “neonatal HIE” OR “newborn hypoxic-ischemic encephalopathy” OR “perinatal asphyxia”. The search was restricted to English-language articles. The article type is limited to “Article”, excluding other types such as “Meeting”, “Case report” and “Review article”, in order to ensure that only published articles that have undergone rigorous and systematic scientific review are included, and to avoid the interference of secondary literature. To avoid the effect of database updates, all data results were downloaded on the same day. It is worth noting that as of the execution time of the search (October 15, 2024), although it was not possible to fully incorporate all the published literature in 2024, we still hoped to conduct a reliable and relatively complete quantitative characterization of the status of this research field in 2024. Therefore, the published literature of that year was retained for subsequent analysis. File formats include plaintext, BibTex, and tab-limited files. Finally, 1,165 records were identified for analysis. The detailed search and screening process is shown in Figure 1.

### Data analysis and visualization

In this study, we imported the retrieved data into the bibliometrix (version 4.3.0) package in CiteSpace (version 6.3. R1), VOSviewer (version 1.6.20), R software (version 4.4.0), and KH Coder (version 3b07d) for analysis and visualization. VOSviewer was used to visualize knowledge graphs, including co-occurrence analysis and co-citation analysis of large-scale literature data, representing authors, journals, and other relevant information. It has been widely used in bibliometric analysis and research (5, 6). We also used CiteSpace (version 6.3. R1) to perform reference cluster analysis to identify developments and future trends in the research field (7).

Additionally, an international cooperation network among countries was mapped using an online scientometrics platform. The bibliometrix package in R software was used to generate histograms of citations, illustrating citation relationships between documents and revealing how knowledge is transferred within the field of study. By analyzing the chain of citations, we can trace the spread of important theories or discoveries.

We employed the latent Dirichlet allocation (LDA), a widely-used topic modeling method capable of efficiently processing unstructured textual data (8), to analyze large-scale document collections. Through LDA, we first generated a vocabulary of term functions by statistically analyzing word co-occurrence patterns across documents. We then quantified the association between documents and latent topics based on term frequency distributions within each text.

Raw data downloaded from WoSCC were imported into Microsoft Excel 2021 for the initial collation. Only article titles and abstracts were retained to form the original corpus. To ensure result reliability and validity, word frequency thresholds were established, generic stop words were removed, and specific stop words were defined. LDA-based topic modeling and word cloud mapping were performed using KH Coder software and Word Cloud. At the final stage, research topics were manually labelled based on the 10 most relevant articles and 20 key topic terms.

### Partial language polishing using ChatGPT 4

We used ChatGPT (Open AI, version 4.0) for part of the language polishing. The date of the visit is February 6, 2025. We have manually checked and guaranteed the accuracy of the AI polished content.

### Ethical considerations

This study is a bibliometric analysis and therefore ethical approval is not applicable.

## Results

### Annual trend of publications

**Figure 2:**
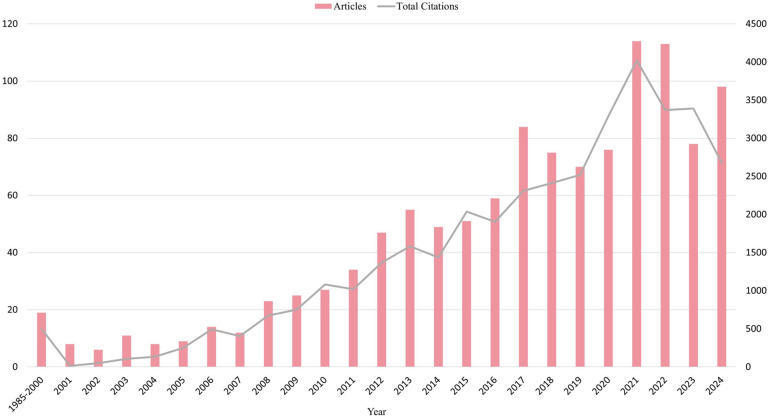
Annual trends in publications and citations. The blue strips represent articles and the red lines represent citations. The horizontal coordinate represents the year, the left vertical coordinate represents the number of articles and the right vertical coordinate represents the total number of citations.

Our analysis of the annual number of publications and citations in the field from 1985 to 2024 (as of the time of the search execution: October 15, 2024) provides insight into the research trends and the importance of this area. As shown in Figure 2. Between 1985 and 2024, the number of publications and citations in the field of TH in neonatal HIE showed different trends. There is an increasing trend in the number of published papers, indicating that research in this field is gradually gaining attention. The number of citations also increases with the number of published papers, reflecting the high academic value and clinical significance of research results in this area.

### Subject distribution analysis

**Figure 3:**
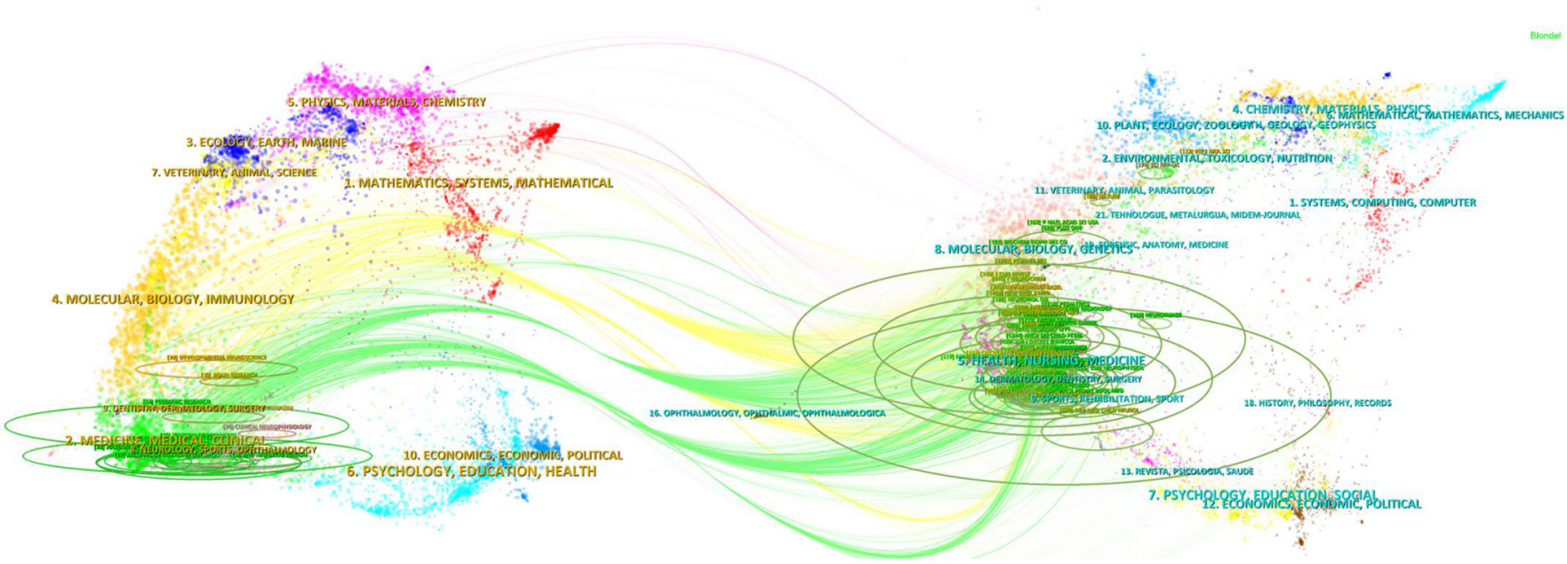
Subject analysis using a double-graph superposition approach. Researches in this area involves many disciplines, showing the characteristics of multi-discipline research.

As illustrated in Figure 3, the subject distribution analysis, based on the superimposed CiteSpace double charts, demonstrates the interdisciplinary nature of research on TH in neonatal HIE. The participation of different disciplines has provided a wealth of perspectives and methods for research in this area, contributing to a more comprehensive understanding and solution for the complex medical problems of neonatal HIE.

### National co-authorship, institutional co-authorship, co-citation and journal co-citation network analysis

**Figure 4:**
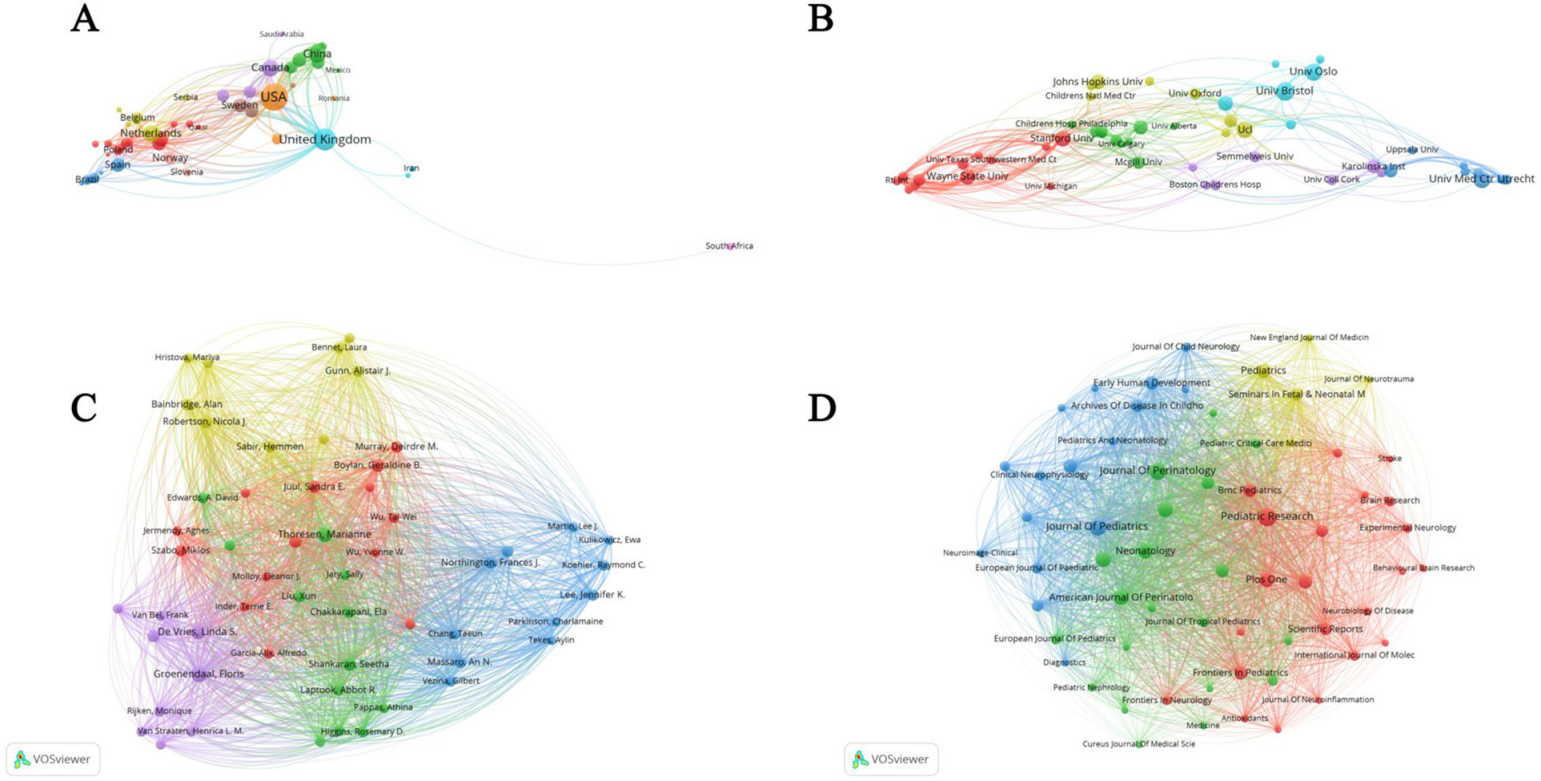
National co-authorship, institutional co-authorship, co-citation and journal co-citation network analysis. **(A)** Analysis of national co-authorship networks; **(B)** institutional co-authorship network analysis; **(C)** author co-citation network analysis; **(D)** journal co-citation network analysis.

Figure 4A highlights significant differences in the number of publications in this field between different countries. The United States had the highest number with 361. Figure 4B shows the differences in the research activities of different institutions in this field. The University of Bristol led this process with 45 publications. Figure 4C shows that highly cited authors, such as Maier, Steven F. Watkins, and Linda R., each with 808 citations, have high visibility and influence in this field. Figure 4D illustrates that of the many journals, the New England Journal of Medicine leads with 4,472 citations.

We also summarized the total number of publications, number of citations, and average number of citations for countries, authors, institutions, and journals, and the results are shown in Table 1.

**Table 1:** Total number of publications, citation times, average citation times of countries, authors, institutions and journals.

Ranking		NC	NP	AC
Journals				
1	New England Journal Of Medicine	4,472	5	894.4
2	Pediatrics	2,730	24	113.75
3	Journal Of Pediatrics	1,936	45	43.0222
4	Pediatric Research	1,529	54	28.3148
5	Archives Of Disease In Childhood-Fetal And Neonatal Edition	1,042	17	61.2941
6	Journal Of Perinatology	927	42	22.0714
7	Neonatology	691	31	22.2903
8	PLos One	671	25	26.84
9	Journal Of Cerebral Blood Flow And Metabolism	602	15	40.1333
10	BMC Pediatrics	589	20	29.45
Country				
1	USA	15,607	361	43.2327
2	United Kingdom	10,489	194	54.067
3	China	1,354	94	14.4043
4	Netherlands	2,789	90	30.9889
5	Canada	4,073	85	47.9176
6	Italy	1,169	68	17.1912
7	Germany	1,122	49	22.898
8	Japan	547	46	11.8913
9	Spain	763	46	16.587
10	Sweden	1,718	46	37.3478
Institutions				
1	Univ Bristol	5,690	45	126.4444
2	Univ Med Ctr Utrecht	1,116	39	28.6154
3	Univ Oslo	1,049	38	27.6053
4	Ucl	3,993	37	107.9189
5	Stanford Univ	4,005	35	114.4286
6	Wayne State Univ	4,499	35	128.5429
7	Johns Hopkins Univ	685	31	22.0,968
8	Univ Auckland	3,402	31	109.7419
9	Univ Calif San Francisco	2,779	28	99.25
10	Karolinska Inst	643	26	24.7308
Authors				
1	Thoresen, Marianne	3,377	35	96.4857
2	Edwards, A. David	2,489	12	207.4167
3	Groenendaal, Floris	1,337	44	30.3864
4	Robertson, Nicola J.	1,248	23	54.2609
5	De Vries, Linda S.	1,242	27	46
6	Azzopardi, Denis	1,230	12	102.5
7	Shankaran, Seetha	1,199	22	54.5
8	Bainbridge, Alan	1,029	16	64.3125
9	Laptook, Abbot R.	871	16	54.4375
10	Liu, Xun	846	15	56.4

NP, number of publications; NC, number of citations; AC, average citations (NC/NP)/.

**Figure 5:**
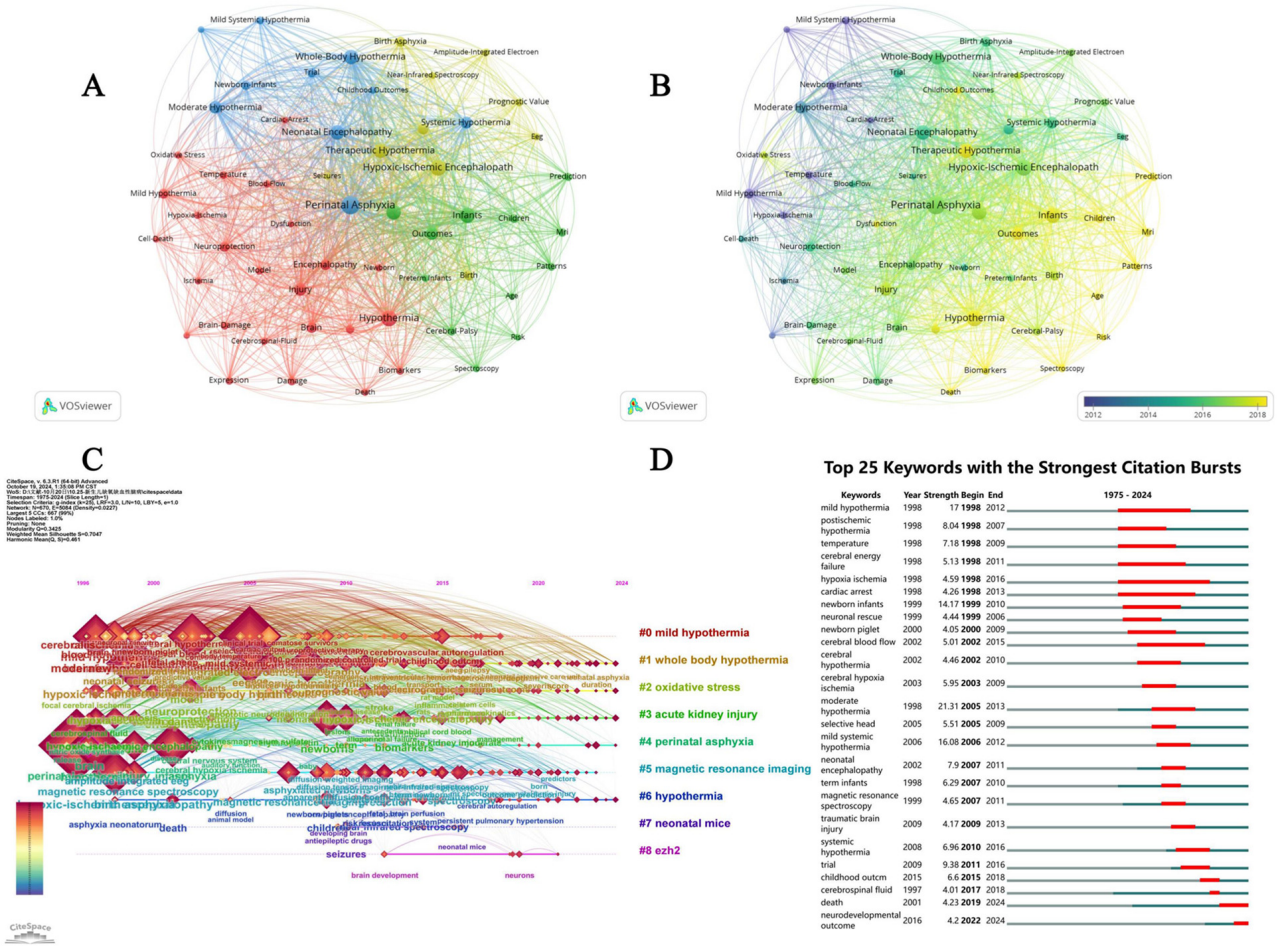
Co-occurrence cluster network, co-occurrence time dimension network, cluster timeline view and citation burst analysis. **(A)** Cluster network analysis based on VOSviewer keyword co-occurrence. The size and color of the nodes represent the strength of the relationship between the frequency of keyword occurrence and the research topic. **(B)** Temporal dimension network analysis of keyword co-occurrence based on VOSviewer. The color of the nodes reflects the research activity of the keywords in different periods. Blue and green represent earlier studies. Red and yellow represent recent research. **(C)** Citespace keyword clustering timeline view. The redder the clustering label color, the closer the study time is to the present. **(D)** Citation burst (top 25 keywords).

In the co-occurrence network shown in Figure 5A, the size and color of the nodes indicate the strength of the relationship between the frequency of keyword occurrences and the research topic. Notable themes included “Mild Systemic Hypothermia”, “Birth Asphyxia”, “amplitude-integrated” “electroencephalography”, “Near-infrared Spectroscopy”, “Childhood Outcome”, and “Prognostic Value”.

Time-dimensional network analysis of keyword co-occurrence using VOSviewer revealed the evolution of these research topics over different periods. Earlier studies, shown in blue and green, focused on topics such as “Therapeutic Hypothermia” and “Hypoxic-Ischemic Encephalopathy.” Over time, the research focus gradually shifted to more specific topics, such as “Mild Systemic Hypothermia,” “Oxidative Stress” and “Biomarkers,” indicating that they have become research hotspots in recent years. Further details are shown in Figure 5B.

The red color of the cluster labels in Figure 5C indicates that the study time was closer to the present time. Using CiteSpace cluster analysis, we can clearly observe research hotspots and trends in the TH of HIE.

Figure 5D shows the top 25 keywords with the strongest citation bursts between 1985 and 2024, with “mild hypothermia” beginning to show citation bursts in 1998 and continuing to 2012 with a higher intensity. This suggests that mild TH has received widespread attention in this field, likely because it can reduce side effects, while still playing a role in the treatment of neonatal HIE.

In addition, after analyzing the occurrence frequency of keywords and their Total Link Strength value, it was found that research on the TH of HIE focused on perinatal asphyxia, hypoxic ischemic encephalopathy, different hypothermia treatment methods, therapeutic effects, prognosis evaluation, the particularity of the study object, and the application of detection technology. Further details are presented in Table 2.

**Table 2:** Keyword occurrence frequency and total association strength value.

Rank	Keyword	Total link strength	Occurrences	Rank	Keyword	Total link strength	Occurrences
1	Perinatal asphyxia	1,672	426	21	Birth	238	57
2	Hypoxic-ischemic encephalopathy	1,160	279	22	Mild systemic hypothermia	202	40
3	Neonatal encephalopathy	992	219	23	Patterns	200	41
4	Brain-injury	907	216	24	Children	193	50
5	Whole-body hypothermia	897	202	25	Prediction	193	40
6	Infants	829	224	26	Cerebral-palsy	183	46
7	Therapeutic hypothermia	787	202	27	MRI	176	42
8	Hypothermia	776	282	28	Prognostic value	174	38
9	Moderate hypothermia	635	146	29	Biomarkers	150	37
10	Outcomes	559	144	30	Asphyxia	147	48
11	Systemic hypothermia	501	109	31	Temperature	147	34
12	Injury	439	128	32	EEG	139	32
13	Term infants	379	79	33	Damage	137	46
14	Birth asphyxia	313	65	34	Childhood outcomes	133	28
15	Neuroprotection	302	69	35	Model	129	34
16	Brain	281	77	36	Amplitude-integrated electroencephalography	128	26
17	Mild hypothermia	268	70	37	Spectroscopy	125	31
18	Encephalopathy	258	82	38	Blood-flow	119	27
19	Trial	248	52	39	Newborn	115	31
20	Newborn-infants	245	58	40	Selective head	115	23

### Citation analysis based on VOSviewer

**Figure 6:**
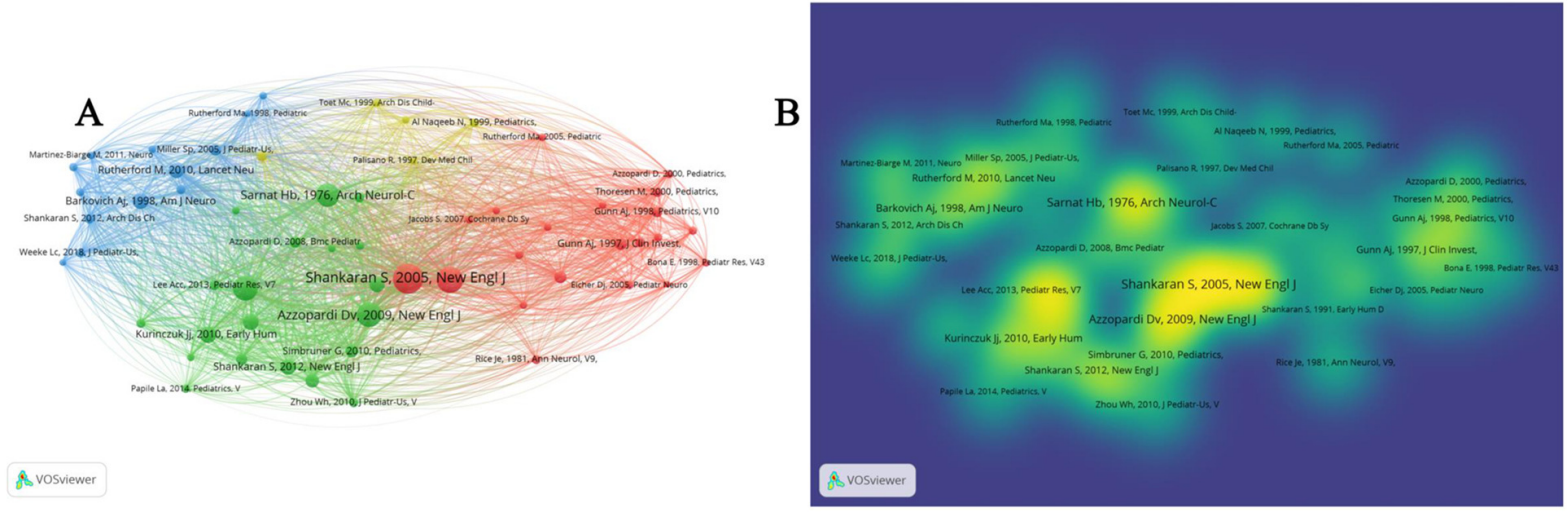
Citation analysis based on vosviewer. **(A**,**B)** Key literature and author network in the field.

VOSviewer's citation analysis in Figure 6 revealed key literature and author networks in the field, where, Toet et al. (48) published in Arch Dis Child Fetal Neonatal Ed, Rutherford, Rutherford et al. (49) published in Lancet Neurol (9), Shankaran et al. (50) published in Arch Dis Child Fetal Neonatal Ed (10), etc. have been frequently cited, showing the core position of these studies in the field of TH of neonatal HIE. Early studies, such as that by Sarnat Hb (51), published in Archives of Neurology (11), have constantly innovated research methods. Over time, our understanding of TH in neonatal HIE has continued to expand.

### Topic modeling

**Figure 7:**
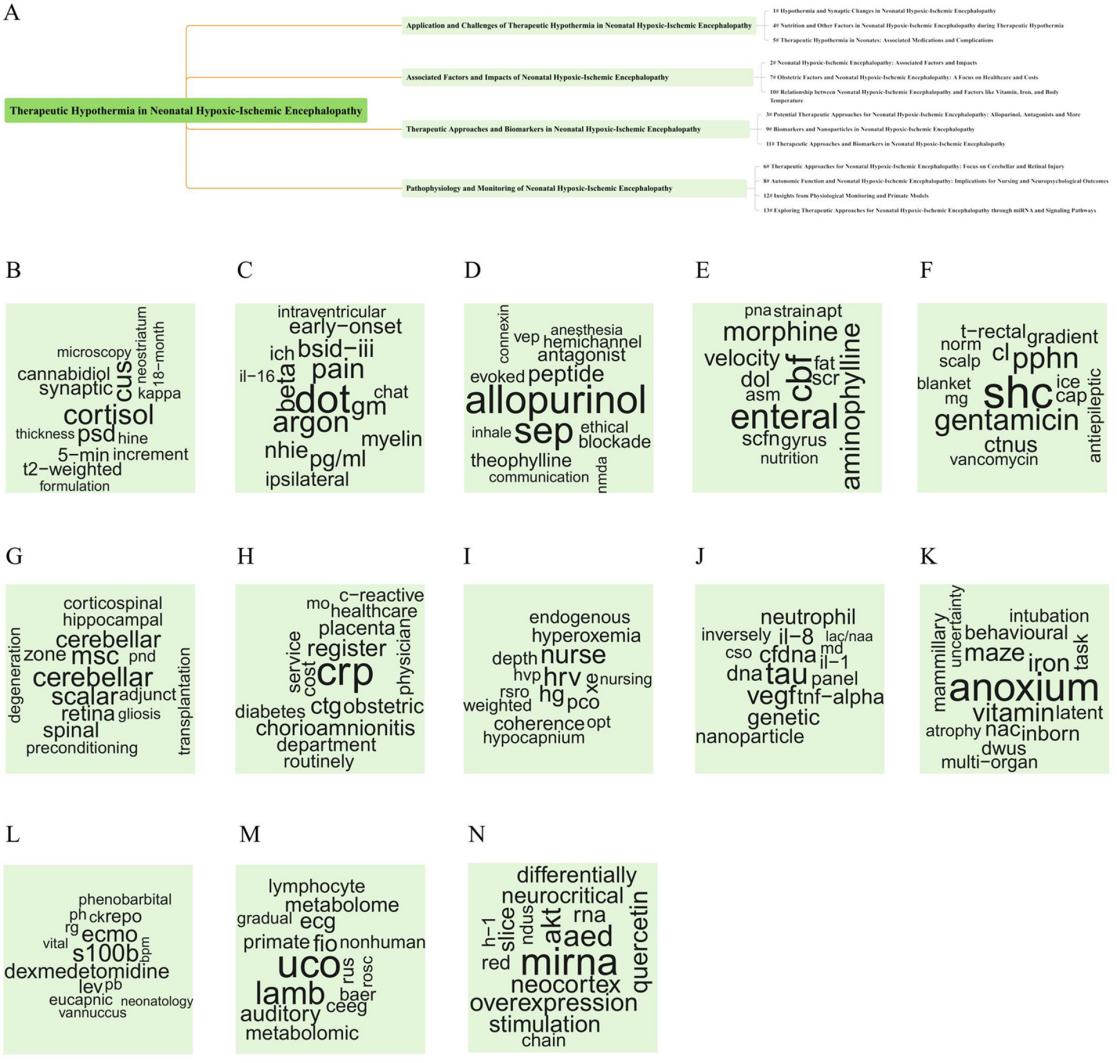
Thematic fishbone map and keyword cloud map based on thematic modeling. **(A**–**N)** used the LDA algorithm to model the topic of 1,165 articles, identified the 13 best topics, classified the content into four main research directions, and summarized the corresponding English topics for each direction.

In this study, the LDA algorithm was used to model the topics of 1,165 articles, identifying 13 key topics based on widely recognized criteria. These topics were further categorized into four main research directions, with corresponding English terms summarized for each direction. These topics included “TH,” “nanoparticle,” “miRNAs” and “signaling pathways,” etc. The results are shown in Figure 7.

## Discussion

We chose bibliometric methods for our study because of their unique advantages over systematic reviews. First, bibliometrics can reveal the development trends and hot topics in a research field through quantitative analysis, thereby providing guidance for future research directions. Second, bibliometrics can identify highly influential authors, institutions and countries, providing essential information for scientific research collaboration. This is often difficult to achieve in systematic reviews. Systematic reviews can delve into specific issues and summarize available evidence, while bibliometrics offers a broader and more dynamic approach to understanding the development of a research field.

Our study identified a significant network of collaborations among authors, institutions, and countries, highlighting the global efforts to address HIE as a critical neonatal condition. In addition, our study has revealed that recent trends have focused on “mild systemic hypothermia,” “oxidative stress,” and “biomarkers”. And we also identified several key research topics through topic modelling, including TH, nanoparticles, miRNAs, and signaling pathways, which are critical for advancing the understanding and treatment of HIE.

### The pathological mechanism of HIE

HIE is a brain damage caused by hypoxia and ischemia that occurs during the perinatal stage and affects the survival and development of newborns. Lack of oxygen impairs the energy metabolism of brain cells, which in turn triggers a chain reaction involving cell damage and death. It has been shown that in the presence of hypoxia, the supply of oxygen to brain tissue is insufficient, resulting in reduced adenosine triphosphate synthesis within cells, damage to cell membrane integrity, and ultimately cell death (12). Hypoxia can trigger a range of inflammatory responses, further exacerbating brain damage (13).

#### Mechanism of oxidative stress in HIE

In the pathogenesis of HIE, oxidative stress is a key factor. The state of hypoxia-ischemia triggers energy depletion, leading to mitochondrial dysfunction and the production of excessive reactive oxygen species (ROS) (14, 15). Continued overproduction of ROS depletes the endogenous antioxidant defense system, making it unable to eliminate harmful free radicals, thereby causing extensive oxidative damage to lipids, proteins, and deoxyribonucleic acid (DNA) (9, 10). Hypoxia and ischemia can also trigger an inflammatory response. Once this inflammatory response is activated, it is combined with oxidative stress to create a vicious cycle of mutual reinforcement (11, 16). Recent evidence also indicates that ferroptosis triggered by the collapse of the antioxidant system (such as glutathione peroxidase 4) is an important mechanism for neuronal death (16, 17). Understanding these mechanisms provides a theoretical basis for developing novel neuroprotective strategies targeting antioxidant pathways (such as activating the nuclear factor erythroid 2-related factor 2 pathway), inhibiting ferroptosis, or combining with existing therapies (such as TH).

#### The role of neuronal and glial cell damage in HIE

The core of neural damage in HIE is a vicious cycle formed by neuronal apoptosis and excessive activation of glial cells. Neuronal death occurs mainly through the apoptotic pathway. It has been found that the p75 neurotrophic factor receptor and neurofilament protein are co-expressed, and that inhibiting Nestin exacerbates apoptosis in neurons; In turn, the Fc fragment of human immunoglobulin IgG1 (p75ECD-Fc) significantly reduces apoptosis in neurons after hypoxia by upregulating the expression of Nestin (18). While the activated microglia release pro-inflammatory factors (such as tumor necrosis factor-α and interleukin-1β), they directly damage neurons through paracrine action (19).

In the early stages of HIE, pro-inflammatory type A1 astrocytes dominate, secreting complement components that exacerbate neuronal damage. Treatment with p75ECD-Fc significantly promotes their conversion into neuroprotective type A2 cells and reduces glial scar formation (18). In the later stages of HIE, M1 type microglia release toxic substances such as nitric oxide and ROS; Treatment with P75ECD-Fc drives the M1 to M2 transition, alleviating neuroinflammation and promoting tissue repair (18, 19). The C-X-C motif chemokine ligand 10 (CXCL10) secreted by microglia guides mesenchymal stem cells (MSCs) to migrate to the brain injury site and promotes repair. Inhibiting the CXCL10- C-X-C motif chemokine receptor 3 (CXCR3) axis will block the localization effect of MSCs (19).

#### The role of molecular signaling pathways in HIE

The nuclear factor κB (NF-κB) acts as a crucial transcription factor and its activation mechanism under hypoxic-ischemic conditions is particularly significant. Hypoxic-ischemia can activate the inhibitor of κB (IκB) kinase complex through Toll-like receptors and tumor necrosis factor receptors, leading to the nuclear translocation of NF-κB (20). Hypoxia, on the other hand, can also increase ROS production. The increase in ROS further promotes the activation of NF-κB. NF-κB binds to the promoter of its target genes in the cell nucleus, promoting the expression of a series of pro-inflammatory factors. These factors play an important role in the pathological course of hypoxia-ischemia (21). Furthermore, under conditions of hypoxia and ischemia, the continuous activation of NF-κB may lead to abnormal regulation of the cell cycle, increase cell death, and thereby aggravate nerve damage (22).

The cross-regulatory mechanism of the mitogen-activated protein kinase (MAPK) signaling pathway has gradually attracted attention in the study of HIE. The MAPK signaling pathway is a large signaling family, with the c-Jun N-terminal kinase (JNK) and extracellular-signal-regulated kinase (ERK) pathways being the two most important branches; The phosphatidylinositol 3-kinase/protein kinase B (PI3K/AKT) pathway is another closely related, frequently synergistic or antagonistic signaling pathway. Studies have found that the activation of the JNK pathway can trigger a series of downstream effects, including the release of pro-inflammatory cytokines, which further activate the NF-κB pathway, forming a positive feedback loop, leading to the intensification of the inflammatory response (23). When the ERK pathway is activated, it can enhance the cell's survival signal through the PI3K/AKT pathway, thus resisting cell death caused by oxygen deprivation. After hypoxic-ischemic injury, ERK activation promotes cell regeneration and repair processes, which are critical for the survival of ischemic neurons (24).

### New biomarkers—microRNAs—play a role in the diagnosis and treatment of HIE

Currently, the research on biomarkers for HIE has shifted from single traditional markers (such as protein markers, cell damage/apoptosis markers, inflammation markers, oxidative stress markers, etc.) to multimodal combinations, and has explored new sources of biomarkers, such as microRNAs (miRNAs).

We have already known that miRNAs are non-coding RNAs composed of 22 nucleotides that regulate gene expression by cutting specific messenger RNAs (mRNAs) or inhibiting their translation. Since miRNAs can regulate more than 60% of protein-coding genes in the human genome, they play a crucial role in key cellular processes, including differentiation, proliferation, growth, and apoptosis (25). Additionally, miRNAs exhibit tissue- and time-specific expression in the nervous system, making them potential biomarkers and therapeutic targets (26).

Overexpression of miR-124 significantly improves the survival of neurons under hypoxic conditions and reduces the number of apoptotic cells (27). Additionally, miR-499-5p plays a protective role against hypoxic-ischemic injury by regulating C-reactive protein expression and inhibiting apoptosis in nerve cells (28). Another study confirmed that miR-363-3p alleviates hypoxic-ischemic injury by targeting DUSP5, demonstrating its potential as a therapeutic target (29). These findings suggest that miRNAs may become a key target in future therapeutic strategies for HIE, contributing to the advancement of personalized medicine.

### Application of TH and mild systemic hypothermia to the treatment of HIE

TH is widely used in the management of HIE, primarily to reduce the metabolic rate of brain cells and mitigate hypoxia-induced cell damage. Studies have shown that Low temperatures can significantly inhibit apoptosis and inflammatory responses, thereby protecting the integrity and function of brain cells. Low temperatures can reduce the concentration of intracellular calcium ions, production of intracellular ROS, and damage caused by oxidative stress in cells (30). In addition, hypothermia reduces inflammation by regulating key signaling pathways such as NF-κB and MAPK signaling pathways, thereby promoting cell survival and functional recovery (31). In animal models, TH has been shown to be effective in alleviating brain tissue damage following hypoxic ischemia, reducing cell mortality, and improving the recovery of neurological function (32).

Mild systemic hypothermia is currently the most widely used and evidence-based treatment method for TH in clinical practice. It refers to a standardized neuroprotective treatment for neonates with moderate to severe HIE. It is initiated within 6 h after birth, using active cooling techniques to precisely control the core body temperature within the range of 33°C–34°C, and maintaining this temperature for 72 h. Subsequently, the body temperature is slowly restored (33).

In clinical applications, TH has been shown to significantly improves the prognosis of HIE. Clinical trials have reported that after 72 h of TH, infants exhibit improved neurological function scores, increased survival rates, and better long-term neurodevelopmental outcomes (34). Additionally, TH has been found to reduce the incidence of epilepsy and the risk of long-term neurological damage in newborns (35). Despite these benefits, the efficacy of TH may be influenced by several factors, including the timing of treatment initiation, the duration of hypothermia, and the overall health status of the newborn (32).

Although TH has shown some efficacy in treating HIE, approximately 29% of newborns treated with this treatment still develop severe neurological developmental disorders (36, 37). Clinical studies have shown that while TH can significantly reduce mortality and the risk of major disability, it cannot completely eliminate the long-term complications associated with HIE, such as epilepsy, motor impairment, and cognitive impairment (14, 38). Several studies have also found that newborns may still experience a persistent neuroinflammatory response even after TH treatment, which may be an important factor in poor outcomes (36, 39). TH may also cause the newborn's body temperature to become too low, thereby triggering complications such as arrhythmia and coagulation dysfunction (31). In low-income countries, TH implementation is even less satisfactory due to the scarcity of medical resources. This further highlights the importance of seeking alternative or complementary treatments (40). Therefore, relying solely on TH is not sufficient to meet the needs of all patients. It is necessary to explore other drugs or interventions to enhance its effectiveness (39).

### Emerging treatments

Emerging treatments for HIE are still in the exploratory stage.

Creatine is an important component of cellular energy metabolism. By participating in the creatine-phosphate system, it helps maintain the level of adenosine triphosphate, thereby supporting neuronal function and reducing damage related to energy depletion (41, 42). Creatine supplementation has been proven to protect brain tissue by reducing oxidative stress, inhibiting inflammatory pathways, and decreasing cell apoptosis (42). However, current data from related studies are still insufficient.

Nanoparticles typically range in size from 1 to 100 nm, which are tiny dimensions that allow them to penetrate cell membranes and enable efficient drug delivery within cells (43).

One study showed that the use of magnetic nanoparticles enabled local heating by an external magnetic field at low temperatures, thus enhancing the efficacy of drugs (44). Therefore, the use of nanoparticles can enhance the drug delivery efficiency at low temperatures and help the drug cluster better in the damaged area. In future, the combination of nanoparticles and TH may produce synergistic effects, providing new ideas and possibilities for the treatment of HIE.

Most emerging therapies are still in the research phase and far from becoming routine clinical treatments.

### Current trends and collaborations play a major role in advancing HIE research

At present, the research on HIE mainly focuses on a series of new targets and emerging treatments, which will promote the early diagnosis of the disease, the precision of treatment, and the assessment of prognosis.

In terms of the practical implications of advancing HIE research, it is crucial to understand how the most active authors or institutions influence future work, collaborations or funding directions. On the one hand, by analyzing bibliometric data, one can identify researchers and institutions with significant influence in this field, thus providing a basis for future collaborations. In addition, these active authors are often able to attract more grant support, thereby facilitating larger scale and more innovative research projects.

### External factors affecting the diagnosis and treatment of HIE

HIE is not merely a medical issue; This is a complex challenge, involving multiple external factors. Among the external factors, underfunding often leads to delays in infrastructure construction (45, 46), while a lack of necessary human resources and training often results in ineffective infrastructure utilization. This, in turn, affects the quality of care (9). In addition, the inadequacy of regulatory policies can also lead to inconsistencies in clinical practice, making it difficult to extend best practices to all medical institutions. Therefore, in order to optimize treatment outcomes for patients with HIE, it is necessary to start from a systemic perspective and consider and continuously improve these key external factors comprehensively to ensure that every newborn receives timely and effective intervention (16, 47).

### Strengths and limitations

The study combines multidimensional bibliometric methods (coauthor/co-occurrence/co-citation analysis) with topic modeling, supported by a robust dataset (1,165 WoSCC records), to systematically map HIE research trends. It bridges theoretical insights (e.g., mild systemic hypothermia, oxidative stress, biomarkers and signaling pathways) to clinical priorities, emphasizing actionable strategies for optimizing TH in neonatal care.

However, this study has some limitations that warrant consideration. First, the research relied primarily on bibliometric analyses and lacked integration with wet-lab experiments, which could provide a more comprehensive understanding of the biological mechanisms underlying TH in neonatal HIE. Second, the sample size of the reviewed literature, although substantial, may not have encompassed all relevant studies, potentially leading to a skewed understanding of the field. Third, this study included only original articles published in English, which may have led to the omission of key regional studies published in non-English languages. There is a possibility that the data set over-represents the perspectives and research activities in high-income countries and does not fully reflect the true research trends and challenges in the global context, especially in lower-income countries with high HIE burdens. Fourth, relying on citation metrics (such as the number of citations) to assess influence has inherent biases. These metrics mainly reflect visibility and attention within the mainstream (especially English) academic communication system, which itself is biased towards high-income countries and English-language publications. Fifth, reliance on title and abstract analysis may miss key details in full-text articles. Finally, selection bias between databases (e.g., WoS vs. PubMed) affecting data representativeness.

## Conclusion

In conclusion, this study successfully mapped the landscape of TH research in neonatal HIE, highlighting key collaborations, research hotspots, and emerging trends. The use of advanced bibliometric tools and topic modeling has provided valuable insights into the evolution and future directions of this research area. Integrating these findings with those of experimental and clinical studies could enhance their applicability and impact and ultimately contribute to improved therapeutic strategies for neonatal HIE. The insights gained from this study lay a foundation for future research and underscore the importance of interdisciplinary collaboration in advancing the field.

## Data Availability

The raw data supporting the conclusions of this article will be made available by the authors, without undue reservation.
