# Supplementary figures and images for: Paradigm shifts in neonatal hypoxic-ischemic encephalopathy therapeutics: a four-decade bibliometric exploration of emerging therapeutic dimensions (1985–2024)
Source: Front Pediatr. 2025 Jul 28;13:1611345. doi: 10.3389/fped.2025.1611345 (PMC12336269; doi:10.3389/fped.2025.1611345)

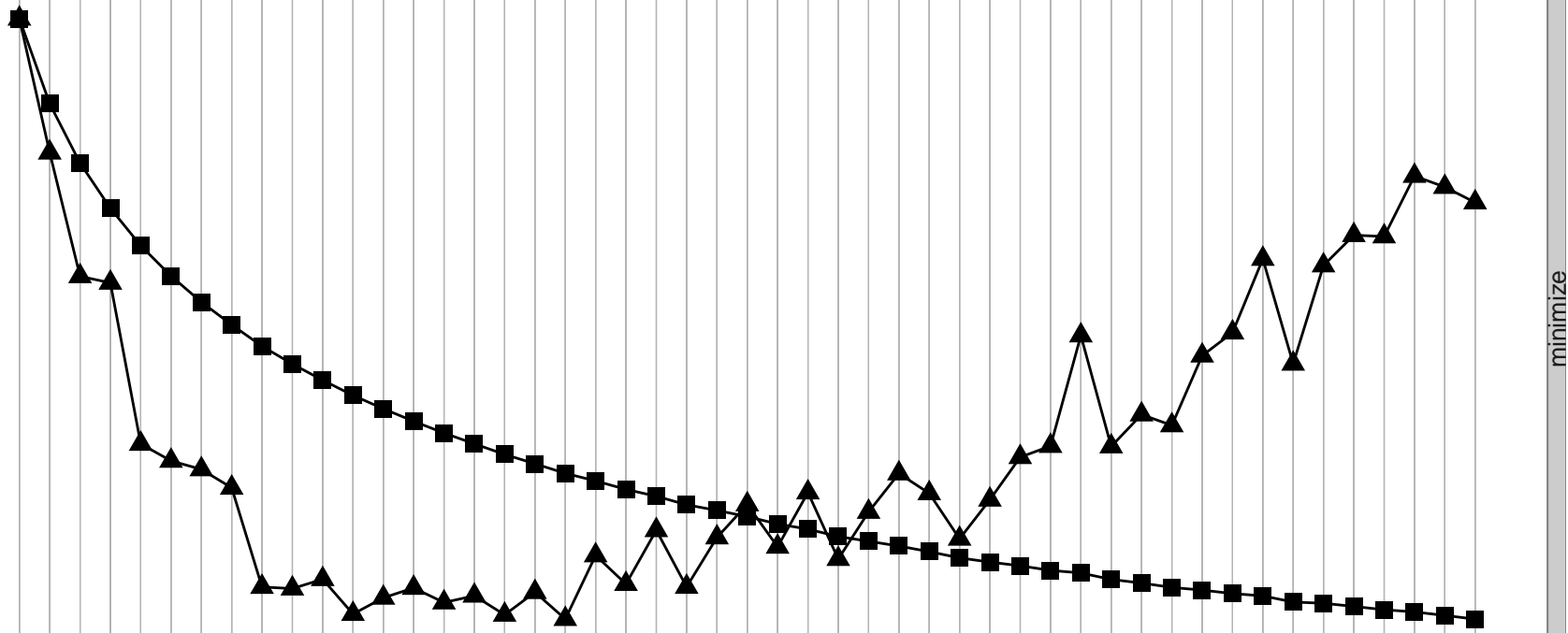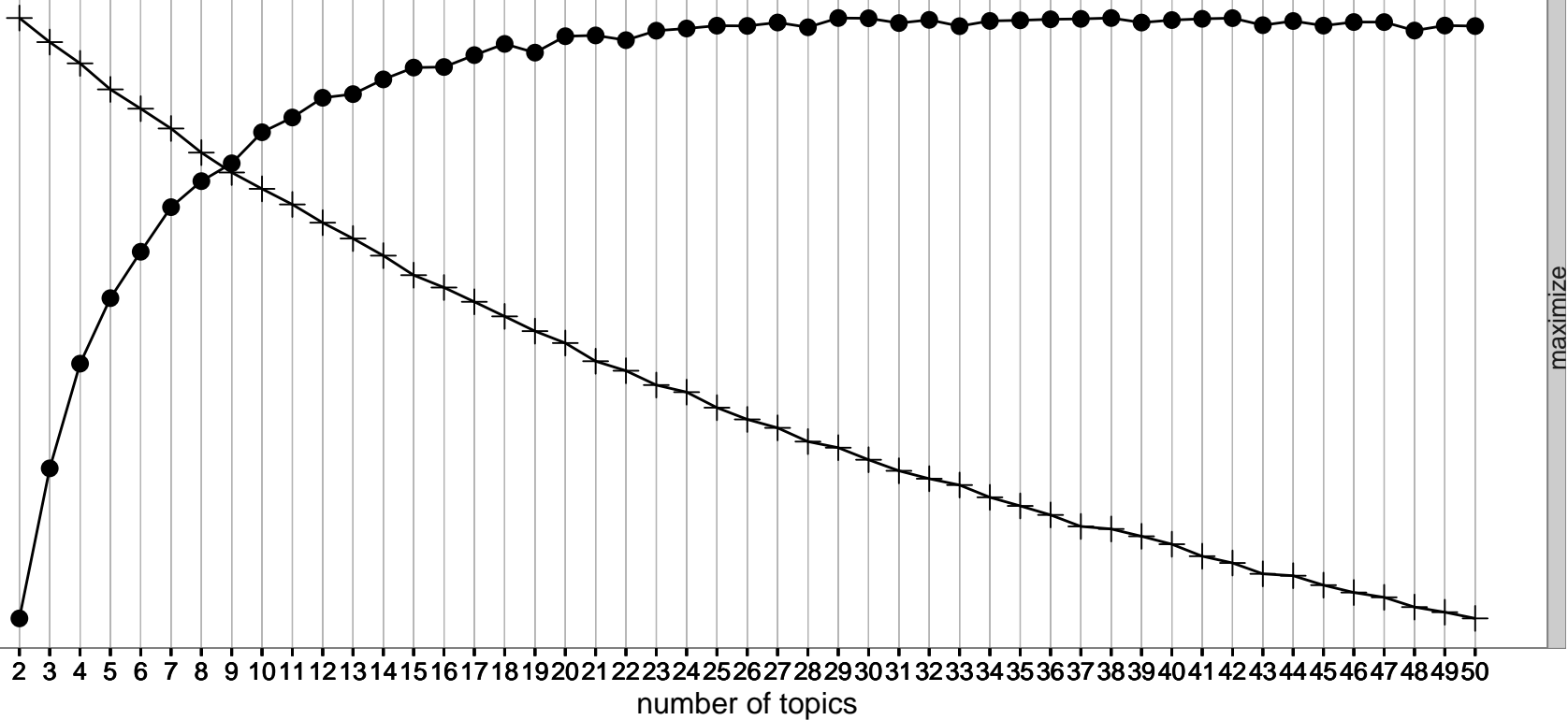

Supplement: Supplementary file 1 [file Datasheet1.pdf]
